# Thyroid Hormones Are Not Associated with Plasma Osteocalcin Levels in Adult Population with Normal Thyroid Function

**DOI:** 10.3390/metabo12080719

**Published:** 2022-08-04

**Authors:** Nikolina Pleić, Dubravka Brdar, Ivana Gunjača, Mirjana Babić Leko, Vesela Torlak, Ante Punda, Ozren Polašek, Caroline Hayward, Tatijana Zemunik

**Affiliations:** 1 Department of Medical Biology, School of Medicine, University of Split, Šoltanska 2, 21000 Split, Croatia; 2 Department of Nuclear Medicine, University Hospital Split, Spinčićeva 1, 21000 Split, Croatia; 3 Department of Public Health, School of Medicine, University of Split, Šoltanska 2, 21000 Split, Croatia; 4 MRC Human Genetics Unit, Institute of Genetics and Cancer, University of Edinburgh, Edinburgh EH4 2XU, UK

**Keywords:** thyroid-stimulating hormone, thyroid hormones, osteocalcin, bone mineral density, body mass index

## Abstract

Thyroid hormones (THs) play an indispensable role in skeletal development and bone remodeling. Some studies have reported associations of THs with serum osteocalcin (OC) levels, but the results are quite inconsistent and the molecular mechanism of their simultaneous or interdependent activity on bone is almost unknown. Therefore, the aim of this study was to determine the possible associations of plasma THs with plasma OC levels and the possible mediating effect of OC on the relationship between THs and bone mineral density (BMD). For this purpose, out of the initial 1981 participants, we selected healthy euthyroid participants controlled for available confounding factors that can affect thyroid function and bone metabolism (*N* = 694). Given our results, we could not confirm any associations of THs with plasma OC levels nor the mediating effect of OC on the relationship between THs and BMD in euthyroid population. In the group of women controlled for menopause status (*N* = 396), we found a significant negative association of body mass index (BMI) with OC levels (β = −0.14, *p* = 0.03). We also found a negative association of free triiodothyronine (fT3) (β = −0.01, *p* = 0.02) and age (β = −0.003, *p* < 0.001) with BMD, and a positive association of BMI (β = 0.004, *p* < 0.001) and male gender (β = 0.1, *p* < 0.001) with BMD. In addition, we found significantly higher plasma OC levels and lower values of BMD in postmenopausal euthyroid women compared with premenopausal euthyroid women. In our opinion, the results of previous studies suggesting an association between circulating THs and serum OC levels may be influenced by an inconsistent selection of participants and the influence of confounding factors.

## 1. Introduction

Bones are a target tissue for a number of hormones, including thyroid hormones (THs) [1]. THs play an irreplaceable role in skeletal development, linear growth, and preservation of bone mass [2]. As early as 1891, Von Recklinghausen was the first to report a patient with hyperthyroidism accompanied by multiple fractures [3]. The clinical impact of hyperthyroidism on the skeleton has been known for a very long time, but the molecular mechanism of THs’ effect on bone is still insufficiently known. In patients with hyperthyroidism, the duration of bone remodeling is reduced with an increase in bone turnover, resulting in decreased bone mineral density (BMD) and a high predisposition for fractures [3,4,5]. On the other hand, in patients with hypothyroidism, the duration of bone remodeling is prolonged leading to low bone turnover and increased BMD [3]. Thyroid-stimulating hormone (TSH) receptors are located in osteoclasts and osteoblasts, and therefore TSH has the potential for bone remodeling independent of THs. Concentrations of TSH showed different correlations with the bone remodeling in men and women. In a group of older men with subclinical thyroid dysfunction, no association of THs with bone turnover markers or fractures was observed [6]. At the same time, a positive association between TSH concentrations and the fracture risk assessment tool (FRAX) score was observed in men, but not in women [4]. In addition, TSH receptor knockout mice who have been given TH replacement therapy failed to reverse the osteoporotic phenotype [7].

The thyroid gland secretes a large amount of thyroxine (T4) and a small amount of bioactive triiodothyronine (T3). T3 is mainly produced by the deiodination of T4 in peripheral tissue. The concentration of T3 in circulation is lower than T4 and has a short half-life. Circulating T4 and T3 are mainly bound to transport proteins, with only a small proportion of circulating free thyroxine (fT4) and free triiodothyronine (fT3) [5]. T3 activity in the cell is mediated by its binding to nuclear T3 receptors where they regulate transcription of the target genes. The T3 receptors (TRα and TRβ) are expressed in osteoblasts and T3 stimulates their activity and differentiation. Among other markers, T3 in osteoblasts promotes osteocalcin (OC) expression [3,5]. T4 has a 100-fold lower affinity for receptor binding than T3 and does not enter the nucleus in sufficient concentrations [3]. Thyroid dysfunction followed by elevated fT4 and low fT3 levels may promote the binding of T4 to the nuclear receptor and its biological activity in the osteoblasts. This explanation suggests that T4 may affect bone homeostasis. Moreover, T4 has a significantly lower effect on OC expression suggesting its different effect on gene expression [5]. In addition to genomic mechanisms, THs also have a nongenomic mechanism in bone cells and T4 may have important nongenomic activity [5].

Bones are also thought to be an endocrine system that produces several hormones which bind to extra-skeletal receptors. In this context, OC synthesized in osteoblasts has a central position [1]. OC undergoes posttranslational modification performed by vitamin K-dependent γ-carboxylase to produce the Gla-protein. OC molecules can be released into circulation both during osteoblastic bone formation and during osteoclastic bone resorption [8]. Therefore, serum OC levels have widely been used as a marker of bone turnover. It has been shown that OC deficient mice present increased bone formation with no effect on bone resorption [9]. This fact indicates that OC is an important factor in bone formation [10,11]. Additionally, apart from bone remodeling, OC is involved in multiple physiological processes. In the pancreatic beta cells, OC activates insulin synthesis and increases insulin sensitivity in the liver, muscles, and fat. OC showed an inverse correlation with glucose metabolism parameters and adiposity. Adipocyte-secreted leptin affects bone formation by inhibiting osteoblast activity and OC synthesis [1].

In the last few years, it has been a particular challenge for the scientific community to understand the association between serum TH levels and serum OC levels. Most of the studies conducted their investigation in differently selected study groups composed of insufficiently selected participants in which many confounding factors can affect bone remodeling and the inferred results. In this study, we analyzed the association between plasma TH and OC levels in an euthyroid population controlling for the influence of most confounding factors that may affect TH levels and bone metabolism. We also analyzed the possible mediating effect of OC on the relationship between THs and BMD.

## 2. Materials and Methods

### 2.1. Study Population

This cross-sectional study initially involved 1981 participants originating from two Croatian cohorts: the mainland city of Split and the island Korcula, derived from the “10 001 Dalmatians project” [12], which is a part of the Croatian Biobank program. Individuals who self-reported thyroid disorder, individuals taking thyroid medication or who underwent thyroid surgery, as well as individuals with TSH, fT3, fT4, thyroglobulin antibodies (TgAb), thyroid peroxidase antibodies (TPOAb), PTH, CT or total serum calcium levels outside of the normal reference range for our population were excluded. We additionally excluded individuals based on a valid history of malignancy, anemia, renal or hepatic dysfunction, sex hormone replacement therapy, and the use of medications that affect bone metabolism. The final number of participants for the analysis was 694. Written informed consent was obtained from participants and the study protocol was approved by the Ethical board of the University of Split, School of Medicine (No: 2181-198-03-04-14-0031 and 2181-198-03-04-19-0022).

### 2.2. Biochemical and Body Measurements

Circulating thyroid/parathyroid hormones/antibodies, CT, and OC in the plasma of individuals were determined by two methods: immunoassay for TSH, fT3, fT4, TPOAb, TgAb, and radio-immunoassay (RIA) for PTH, CT, and OC. Immunoassay reaction was conducted in a fully automated instrument “Liaison XL” Biomedica Chemiluminescence Analyzer (DiaSorin, Saluggia, Italy). RIA is a manual method that runs on the Scintillation counter of liquid samples, Capintec, and 125I serves as a marker. Reference ranges for our study population were: TSH 0.3–3.6 mIU/L, fT3 3.39–6.47 pmol/L, fT4 10.29–21.88 pmol/L, TgAb 5–100 IU/mL, and TPOAb 1–16 IU/mL, PTH 12.26–35.5 pg/mL, CT 0–20 ng/mL and OC 5–25 ng/mL. All biochemical measurements were performed in the Biochemistry Laboratory of the Department of Nuclear Medicine at the University Hospital Split.

Additionally, in our analysis, we included data on BMD, total serum calcium, and BMI (calculated as body weight divided by the squared value of body height) which were obtained during initial participant recruitment. Reference ranges for our study population were: total serum calcium 2.14–2.53 mmol/L, and BMI 18.5–24.9.

Absolute BMD (g/cm^2^) was determined by dual-energy X-ray absorbitary (DEXA) in the heel (CalcScan) using the DEXA bone densitometer DXL produced by Demetech (Stockholm, Sweden). DEXA is a non-invasive method of measuring bone density. X-ray beams of two different energies pass through the bone (heel) and the amount that is not absorbed is detected on the other side of the bone. Bones with higher mineral content absorb more energy, and consequently less energy is detected. The application of two energy beams allows the assessment of absorption from soft tissue separately from bones.

### 2.3. Statistical Analysis

We tested the associations between TSH, fT3, fT4, OC, BMI, and BMD levels using regression models adjusted for known confounders: age and gender. The family wise error rate (FWER) was controlled using the Bonferroni–Holm method. Additionally, to perform the mediation analysis, simple linear regression models were used to assess the association of BMD with THs and TSH and the association of OC with BMD. Independent samples *t*-test was used for comparison of plasma OC, TSH, fT3, fT4, BMI, age, and BMD between the groups of premenopausal and postmenopausal women. Two multiple linear regression analyses were performed to assess the individual effects of gender, age, TSH, fT3, fT4, BMI, and BMD on plasma OC levels, as well as the effect of gender, age, TSH, fT3, fT4, BMI, and OC on BMD levels. Additionally, the two regression models were also fitted in the subgroups of men and women, whereas in the group of women the regression models were additionally controlled for menopause status. Regression assumptions, namely linearity of the data, normality of residuals, and homoscedasticity were checked using diagnostic plots. The level of statistical significance was set at 0.05. Statistical analyses were performed using R: A language and environment for statistical computing (R Foundation for Statistical Computing, Vienna, Austria) [13]. Visualization of the multiple linear regression model coefficients was performed using the “ggstatsplot” R package [14].

## 3. Results

After fulfilling all of the exclusion criteria, the initial number of 1981 participants was reduced to the final 694 healthy euthyroid participants. There were 396 women and 298 men, with a mean age of 51.6. The characteristics of the study participants are shown in Table 1. Continuous variables are expressed as means with standard deviations or as medians with lower and upper quartiles, and categorical variables as frequencies (percentages) (Table 1). The distribution of TSH, fT4, and OC levels was right-skewed, while levels of fT3, fT3/fT4, age, body mass index (BMI), and BMD followed an approximately normal distribution. Plasma OC levels had a median (interquartile range) of 15.6 (13–18.5) ng/mL.

After adjusting the linear regression models for age and gender and applying the Bonferroni–Holm correction (Table 2), fT3 was positively associated with fT4 (*p* < 0.001) and fT3/fT4 ratio (*p* < 0.001), and fT4 was negatively associated with fT3/fT4 ratio (*p* < 0.001). BMI was positively associated with BMD (*p* < 0.001).

Additionally, simple linear regression (without controlling for age and gender) showed that there was no significant association between thyroid hormones and BMD (β_fT3_ = −0.004, p_fT3_ = 0.9; β_fT4_ = 0.05, p_fT4_ = 0.181; β_TSH_ = −0.04, p_TSH_ = 0.32), nor between OC and BMD (β = −0.06, *p* = 0.131). Therefore, with our statistical mediation analysis, we could not confirm the mediating effect of OC on the relationship between thyroid hormones and BMD.

**Figure 1:**
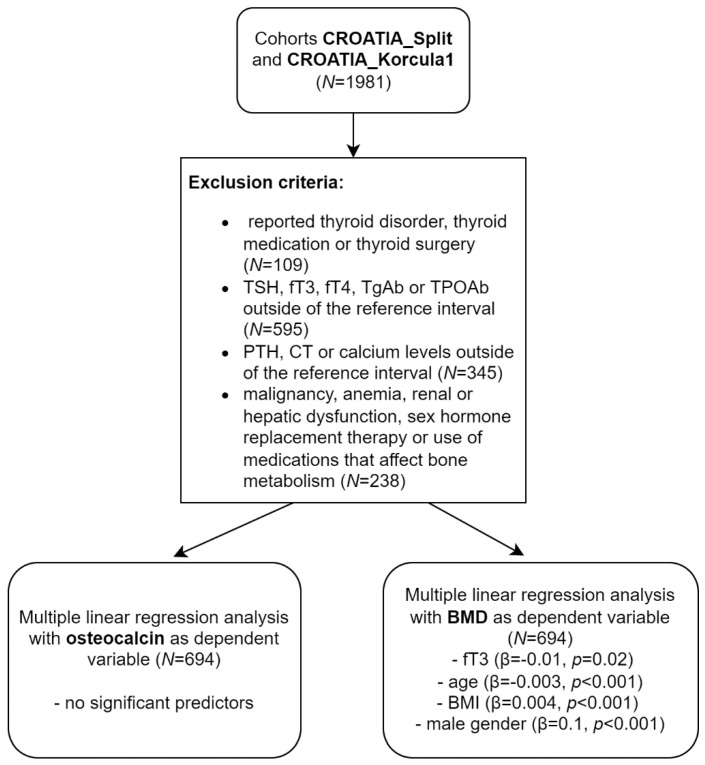
Exclusion criteria to define population and results of linear regression analyses.

Furthermore, two multiple regression models were used to assess the effect of gender, age, BMI, TSH, fT3, and fT4 on BMD and OC levels (Figure 1).

**Figure 2:**
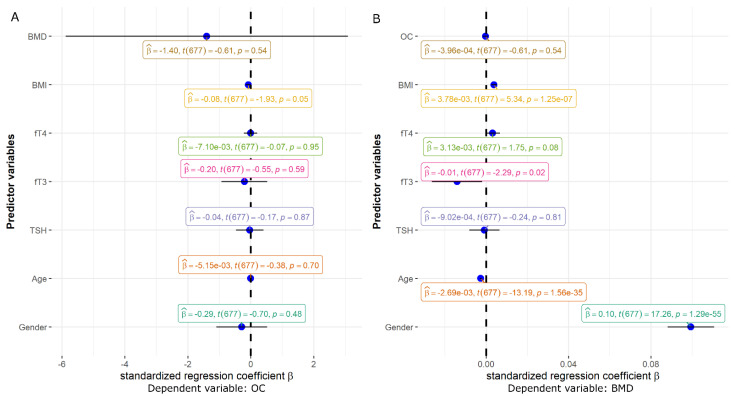
(**A**) Effect sizes and 95% confidence intervals obtained from the linear regression analysis for the association of gender, age, TSH, fT3, fT4, BMI, and BMD with OC plasma levels. (**B**) Effect sizes and 95% confidence intervals obtained from the linear regression analysis for the association of gender, age, TSH, fT3, fT4, BMI, and OC with BMD. Statistically significant predictor variables are the lines that do not intersect with the vertical line at 0. If the lower limit of the confidence interval is above 0, the association is positive, whereas if the upper limit of the confidence interval is below 0, the association is negative.

The first model with OC as a dependent variable showed no significant predictors (Figure 2A). In the second regression model with BMD as a dependent variable (Figure 2B), gender (coded as 0/1 for woman/man) and BMI were positively associated with BMD (both *p* < 0.001). Age (*p* < 0.001) and fT3 (*p* = 0.02) were negatively associated with BMD (Figure 2B).

Furthermore, we divided women in our sample into two groups, premenopausal and postmenopausal (Table 3). There were 396 women, out of which 199 were premenopausal and 197 postmenopausal.

**Figure 3:**
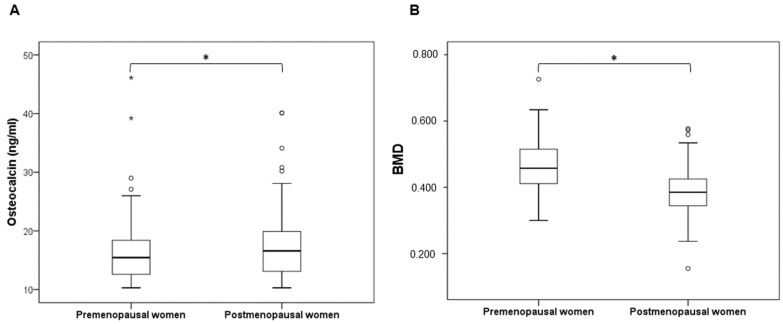
Box plots of (**A**) OC and (**B**) BMD levels in premenopausal and postmenopausal euthyroid women. * Denotes a significant *p*-value of a *t*-test at the significance level of 0.05. Outliers are marked with circles, while extreme outliers are marked with asterisks.

Postmenopausal euthyroid women had significantly higher values of BMI compared to premenopausal women (t = −8.88, df = 391, *p* < 0.001). Postmenopausal euthyroid women had significantly higher levels of plasma OC compared to premenopausal euthyroid women (t = −2.019, df = 392, *p* = 0.044) (Figure 3A). Additionally, BMD levels were significantly lower in postmenopausal euthyroid women compared to premenopausal euthyroid women (t = 10.88, df = 385.6, *p* < 0.001) (Figure 3B).

In addition, given that levels of OC differed significantly between premenopausal and postmenopausal women, the first linear regression model with OC as a dependent variable was fitted separately in men (Supplementary Figure S1) and women (Supplementary Figure S2), and the latter one in the group of women was additionally controlled for menopause status (Supplementary Figure S3). Subgroup analysis revealed no new associations except for the significant negative association of BMI with OC levels (β = −0.14, *p* = 0.03) in the group of women controlled for menopause status (*N* = 396) (Supplementary Figure S3).

Analogous to the OC analysis, given that measures of BMD differed significantly between premenopausal and postmenopausal women, the second linear regression model with BMD as a dependent variable was fitted separately in men (Supplementary Figure S4) and women (Supplementary Figure S5), and the latter one in the group of women was additionally controlled for menopause status (Supplementary Figure S6). The negative coefficient of association between fT3 and BMD remained negative both in the group of men and women, but with very wide confidence intervals meaning that the sample sizes of the subgroup analyses were too small to achieve statistical significance.

## 4. Discussion

Although the main goal of our study was to determine the associations of circulating THs with plasma OC levels and the potential mediating effect of OC on the relationship between THs and BMD, we did not record any association in that sense. Neither gender, age, TSH, fT3, fT4, BMI nor BMD were predictive of plasma OC levels in our euthyroid population. However, in women, when the association was controlled for menopause status, we observed a significant negative association between BMI and plasma OC levels. The next significant finding of our study is a negative association between plasma fT3 levels and BMD, as well as a negative association between age and BMD in well-selected euthyroid participants. When the analysis is performed for men and women separately, the association between fT3 and BMD is no longer significant at the 5% significance level. However, the slight increase in the *p*-value is probably due to the smaller sample size in the separate gender groups. Another significant result is a positive association between BMI and BMD, and a positive association between the male gender and BMD. In addition, BMD values were significantly lower and plasma OC levels were significantly higher in postmenopausal euthyroid women compared to premenopausal euthyroid women.

There are only three studies analyzing associations of THs with serum OC performed so far. The most recent one performed on 1152 euthyroid participants found a positive association of fT3 and fT3/fT4 with OC levels, while BMI was negatively associated with OC levels. This negative association was observed in the total sample comprised of both men and women, while in our study this association became significant only in the case of women controlled for menopause status. They also showed that fT3 and fT3/fT4 suppressed the negative association between BMI and OC levels [15]. A large German population-based study found only a non-linear U-shaped association between TSH and OC levels in women, while a large Australian study performed in older men found no relationship between TSH and fT4 with OC levels [4,6]. Although all three studies were performed in large samples, the results are very divergent. The reasons for this are probably the inconsistent selection of participants and inequality in the exclusion criteria. In our study, we started with a very large sample size, but after rigorous exclusion criteria according to all available parameters that may affect THs’ levels and bone metabolism, the sample was significantly reduced to 694 participants. We are the only study that excluded participants according to the complete thyroid status (including all thyroid hormones and thyroid antibodies), a valid history of malignancy, anemia, renal or hepatic dysfunction, sex hormone replacement therapy, use of medications that affect bone metabolism, and those with parathyroid hormone (PTH), calcitonin (CT) and total serum calcium levels outside of the referent ranges. Although the confounding effects can never be fully eliminated in observational studies, we believe our rigorous selection of participants makes us a step further in inferring the investigated associations.

The results of the studies investigating the impact of thyroid function on BMD are still controversial. Existing studies often give conflicting results due to differences in study design, participant selection, and small sample sizes. A large European study observed that THs, fT4, and fT3 within the upper normal range were associated with reduced BMD [16]. They included 1278 euthyroid postmenopausal women (individuals with thyroid or nonthyroidal illness and those receiving drugs affecting thyroid function or bone metabolism were excluded). This association was observed in the analysis of hip BMD, but not in the analysis of vertebral BMD. Another study performed by Van der Deure et al. included 1151 women and men from the Rotterdam Study, aged 55 years and older (participants were not excluded for nonthyroidal diseases or for taking drugs affecting bone metabolism), and also observed a negative association between fT4 levels and BMD, and positive association between BMD and TSH [17]. The results of these two studies are in accordance with our finding of an inverse association between plasma fT3 levels and BMD. In our study, we analyzed heel BMD. On the other hand, a study performed on 648 postmenopausal Korean women and 728 men showed no association between fT4 and TSH with BMD; however, high–normal fT4 levels were associated with low trabecular bone score (TBS) in postmenopausal women, but not in men [18]. In this study, the authors excluded participants with abnormal thyroid function, those who had undergone thyroid surgery or had thyroid disease, as well as those taking drugs affecting bone metabolism. Furthermore, a recent study showed a positive correlation between TSH and BMD in 114 males with normal thyroid function (participants with chronic, liver, kidney, or other endocrine diseases, as well as those taking drugs that affect bone metabolism, were excluded from the study) [19]. In addition, a Korean study performed on postmenopausal women showed an association between low normal TSH levels and low BMD at the lumbar spine and femoral neck [20]. The authors selected 959 euthyroid postmenopausal women without a history of thyroid disease, a disease affecting bone metabolism, or any other significant disease. Additionally, subjects taking drugs that may affect bone metabolism or thyroid axis were excluded from the study.

We also observed an inverse relationship between age and BMD. It is a common belief that BMD reaches its peak by the age of 35 and then declines gradually [21,22,23,24], and this viewpoint is in some way aligned with our result. Recent studies indicate that TBS may be a better indicator of bone strength changes than BMD because the peak of TBS is reached at the age of about 50 years and has a tendency to fall rapidly from 66 to 75 years of age [22].

Sex hormones in men and women have an important role in the homeostasis of bones. In women, significant bone loss begins at menopause, while in men bone loss progresses gradually with age. Changes in BMD also depend on the site of bone loss or the type of bone in which BMD is determined [22]. Lee et al. described continuous bone loss at the femur with a short-term acceleration phase after the achievement of peak bone mass (PBM) in males. On the other hand, in women, bone loss is divided into three phases: first acceleration phase, consolidation or deceleration phase, and second acceleration phase [22]. Similar bone loss was described by Warming L et al. [25]. In our study, we observed a positive association between the male gender and BMD. Since half of the women in our study group were postmenopausal, women were expected to have lower BMD than men and a positive association between the male gender with BMD was found accordingly.

We also observed a positive association between BMD and BMI. In 1999, Ertungealp et al. found a significant association between BMD and BMI in every location (lumbar vertebrae and in the classical locations of the proximal femur such as the femoral neck, Ward’s triangle, and the trochanter), and in every age group of pre/post-menopausal women in Turkey [26]. Another study from 2012 analyzing clinical parameters that can affect BMD found that BMI was the leading clinical parameter associated with BMD [22]. Thus, the results of our study confirm the previous findings.

Even though we eliminated a large proportion of potential confounding factors, our study still has some limitations. We did not have concentration data of gonadal hormones and vitamin D (1α,25-dihydroxyvitamin D (1,25(OH)2D3)), as additional potential confounding factors that can affect the results of the study. The mediating effect of OC on the relationship between THs and BMD was evaluated only in silico in our cross-sectional data, and further experimental studies need to be conducted to confirm its presence or absence. BMD was evaluated in the heel, and it would have been better to have data for BMD in the spine and femur, which would be easier to compare with published studies. The last limitation of our study is based on the mentioned modern approach in the determination of the TBS, which is considered a better indicator of bone strength changes, as opposed to the BMD measure used in our study.

In conclusion, our data show no association between plasma TH levels and plasma OC levels. Our statistical mediation analysis found no mediating effect of OC on the relationship between THs and BMD. The strength of our study is that the associations were evaluated in rigorously selected participants. Observing the divergent results of similar studies published so far, our study indicates that inadequately selected participants can result in confounded results and conclusions.

## Figures and Tables

**Table 1:** Clinical characteristics of study participants.

Variable	Total (*N* = 694)	Reference Interval
Women	396 (57.1%)	-
Age	51.6 (14.8)	-
OC	15.6 (13–18.5)	5–25 ng/mL
CT	5.2 (2.68–8.2)	0–20 ng/mL
TSH	1.43 (1.05–2.01)	0.3–3.6 mIU/L
fT3	4.57 (0.49)	3.39–6.47 pmol/L
fT4	12.7 (11.9–13.9)	10.29–21.88 pmol/L
fT3/fT4	0.36 (0.05)	-
TgAb	7.6 (5–11.2)	5–100 IU/mL
TPOAb	2.7 (1.3–6.3)	1–16 IU/mL
PTH	21.5 (5.7)	12.26–35.5 pg/ml
Total serum Calcium	2.36 (0.1)	2.14–2.53 mmol/L
BMI	27.31 (4.34)	18.5–24.9
Absolute BMD	0.47 (0.1) (g/cm^2^)	-

Data are presented as mean (standard deviation), median (lower quartile–upper quartile), or frequency (percentage). BMD, bone mineral density; BMI, body mass index; CT, calcitonin; fT3, free triiodothyronine; fT4, free thyroxine; OC, osteocalcin; PTH, parathyroid hormone; TgAb, thyroglobulin antibodies; TPOAb, thyroid peroxidase antibodies; TSH, thyroid-stimulating hormone.

**Table 2:** Association between TSH, thyroid hormones, osteocalcin, BMI, and BMD in the total sample (*N* = 694).

Variable	OC	TSH	fT3	fT4	fT3/fT4	BMI	BMD
OC	-	-	-	-	-	-	-
TSH	−0.01	-	-	-	-	-	-
fT3	−0.02	−0.005	-	-	-	-	-
fT4	−0.004	−0.08	0.28 *	-	-	-	-
fT3/fT4	−0.01	0.07	0.31 *	−0.45 *	-	-	-
BMI	−0.09	−0.023	−0.043	−0.065	0.021	-	-
BMD	−0.05	−0.023	−0.077	0.026	−0.101	0.204 *	-

Effect size estimates of the associations as evaluated by regression analyses adjusted for age and gender. * Denotes a significant *p*-value after the Bonferroni–Holm correction. BMD, bone mineral density; BMI, body mass index; fT3, free triiodothyronine; fT4, free thyroxine; OC, osteocalcin; TSH, thyroid-stimulating hormone.

**Table 3:** Clinical characteristics of premenopausal and postmenopausal women.

Variable	Premenopausal Women (*N* = 199)	Postmenopausal Women (*N* = 197)	*p*-Value
Age	40.2 (10.98)	61.7 (8.5)	<0.001 *
OC (ng/mL)	15.45 (12.6–18.43)	16.6 (13.1–19.9)	0.044 *
TSH (mIU/L)	1.52 (1.15–2.85)	1.41 (1.08–2.01)	0.294
fT3 (pmol/L)	4.6 (0.4)	4.5 (0.48)	0.094
fT4 (pmol/L)	12.7 (11.9–13.9)	12.3 (11.7–13.5)	0.223
BMI	26.64 (4.3)	28.32 (4.0)	<0.001 *
Absolute BMD (g/cm^2^)	0.46 (0.1)	0.39 (0.1)	<0.001 *

Data are presented as mean (standard deviation) or as median (lower quartile–upper quartile). * Denotes a significant *p*-value of a *t*-test at the significance level of 0.05. BMD, bone mineral density; BMI, body mass index; fT3, free triiodothyronine; fT4, free thyroxine; OC, osteocalcin; TSH, thyroid-stimulating hormone.

## Data Availability

The data presented in this study are available on request from the corresponding author. There is neither Research Ethics Committee approval, nor consent from individual participants, to permit the open release of the individual-level research data underlying this study. The datasets analyzed during the current study are therefore not publicly available.

## Supplementary Materials

The following supporting information can be downloaded at: https://www.mdpi.com/article/10.3390/metabo12080719/s1, Figure S1: Effect sizes of the linear regression model with OC as a dependent variable in the group of men; Figure S2: Effect sizes of the linear regression model with OC as a dependent variable in the group of women; Figure S3: Effect sizes of the linear regression model with OC as a dependent variable in the group of women additionally controlled for menopause status; Figure S4: Effect sizes of the linear regression model with BMD as a dependent variable in the group of men; Figure S5: Effect sizes of the linear regression model with BMD as a dependent variable in the group of women; Figure S6: Effect sizes of the linear regression model with BMD as a dependent variable in the group of women additionally controlled for menopause status.

## Abbreviations

1,25(OH)2D3, 1α,25-dihydroxyvitamin D; 25(OH)D, 25-hydroxyvitamin D; BMD, bone mineral density; BMI, body mass index; CT, calcitonin; FRAX, fracture risk assessment tool; fT3, free triiodothyronine; fT4, free thyroxine; FWER, family wise error rate; OC, osteocalcin; PBM, peak bone mass; PTH, parathyroid hormone; RIA, radio-immunoassay; T4, thyroxine; TBS, trabecular bone score; TgAb, thyroglobulin antibodies; THs, thyroid hormones; TPOAb, thyroid peroxidase antibodies; TSH, thyroid-stimulating hormone.
