# Supplementary material for: Thyroid Hormones Are Not Associated with Plasma Osteocalcin Levels in Adult Population with Normal Thyroid Function
Source: Metabolites. 2022 Aug 4;12(8):719. doi: 10.3390/metabo12080719 (PMC9412351; doi:10.3390/metabo12080719)
Supplement: Supplementary file 1 [file metabolites-12-00719-s001.zip › metabolites-1850346-supplementary.pdf]

Supplementary material

Osteocalcin analysis

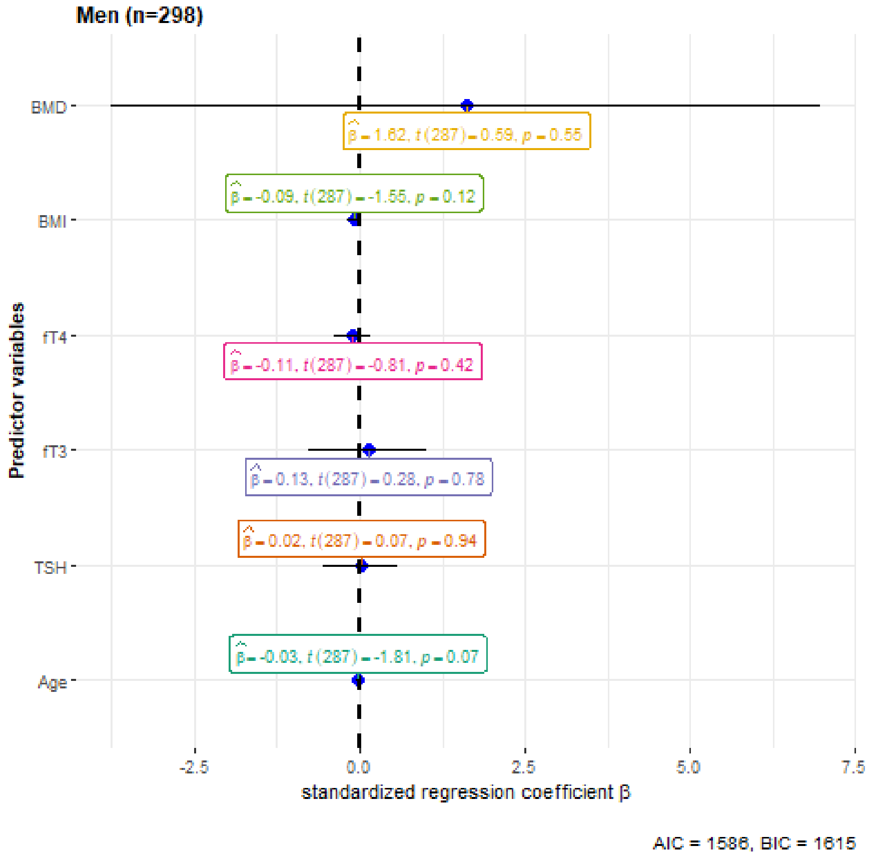

Figure S1. Effect sizes and 95% confidence intervals obtained from the linear regression analysis for the association of age, TSH, fT3, fT4, BMI and BMD with Osteocalcin in the group of men (n=298). Statistically significant predictor variables are the lines that do not intersect with the vertical line at 0. If the lower limit of the confidence interval is above 0, the variable is positively associated with Osteocalcin, whereas if the upper limit of the confidence interval is below 0, the association is negative.

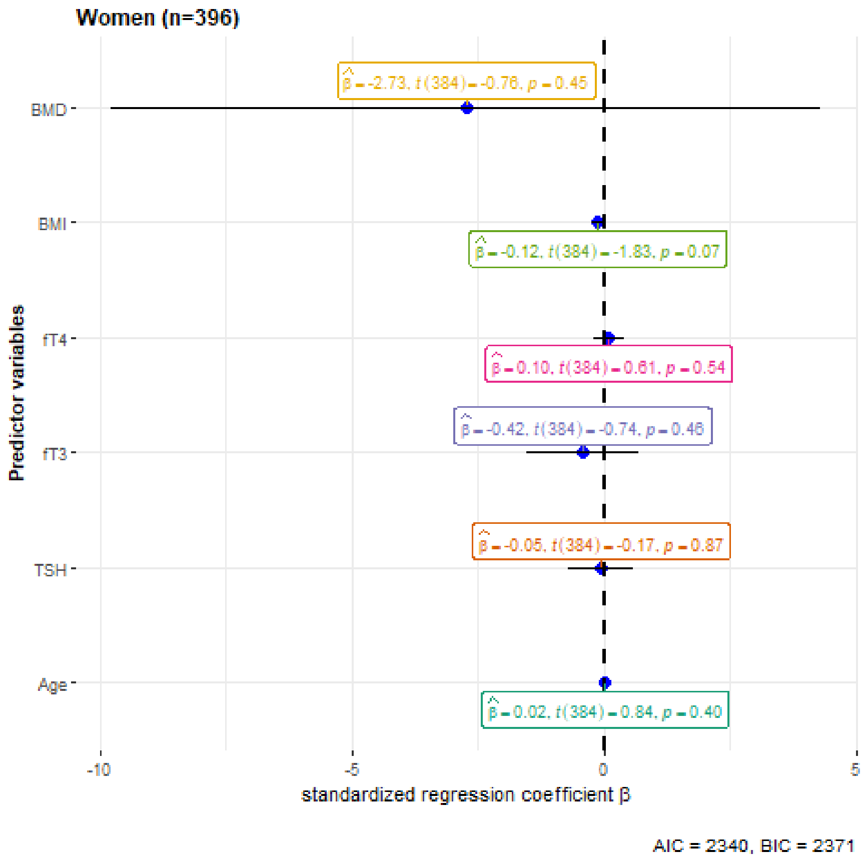

Figure S2. Effect sizes and 95% confidence intervals obtained from the linear regression analysis for the association of age, TSH, fT3, fT4, BMI and BMD with Osteocalcin in the group of women (n=396). Statistically significant predictor variables are the lines that do not intersect with the vertical line at 0. If the lower limit of the confidence interval is above 0, the variable is positively associated with Osteocalcin, whereas if the upper limit of the confidence interval is below 0, the association is negative.

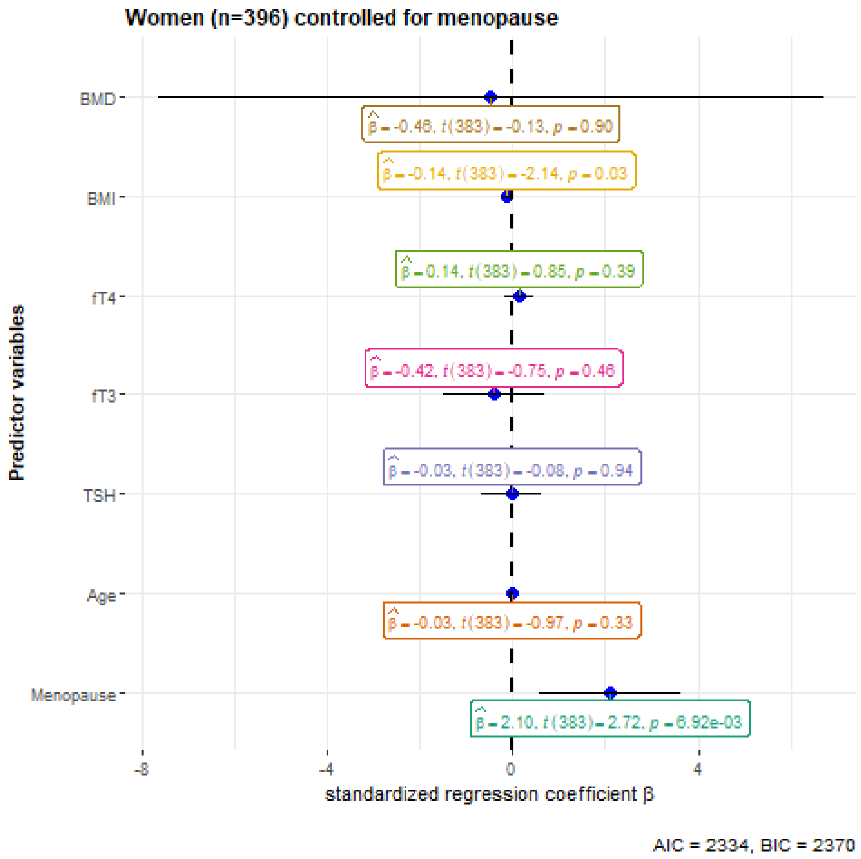

Figure S3. Effect sizes and 95% confidence intervals obtained from the linear regression analysis for the association of menopause status, age, TSH, fT3, fT4, BMI and BMD with Osteocalcin in the group of women (n=396). Statistically significant predictor variables are the lines that do not intersect with the vertical line at 0. If the lower limit of the confidence interval is above 0, the variable is positively associated with Osteocalcin, whereas if the upper limit of the confidence interval is below 0, the association is negative.

Bone mineral density (BMD) analysis

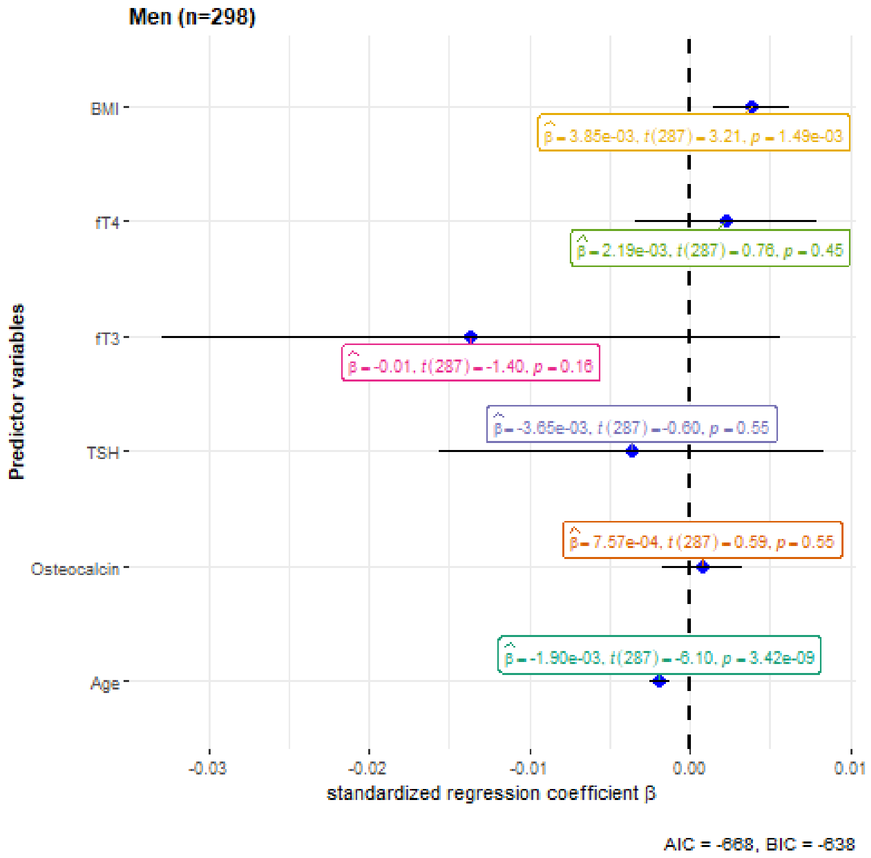

Figure S4. Effect sizes and 95% confidence intervals obtained from the linear regression analysis for the association of age, Osteocalcin, TSH, fT3, fT4 and BMI with BMD in the group of men (n=298). Statistically significant predictor variables are the lines that do not intersect with the vertical line at 0. If the lower limit of the confidence interval is above 0, the variable is positively associated with BMD, whereas if the upper limit of the confidence interval is below 0, the association is negative.

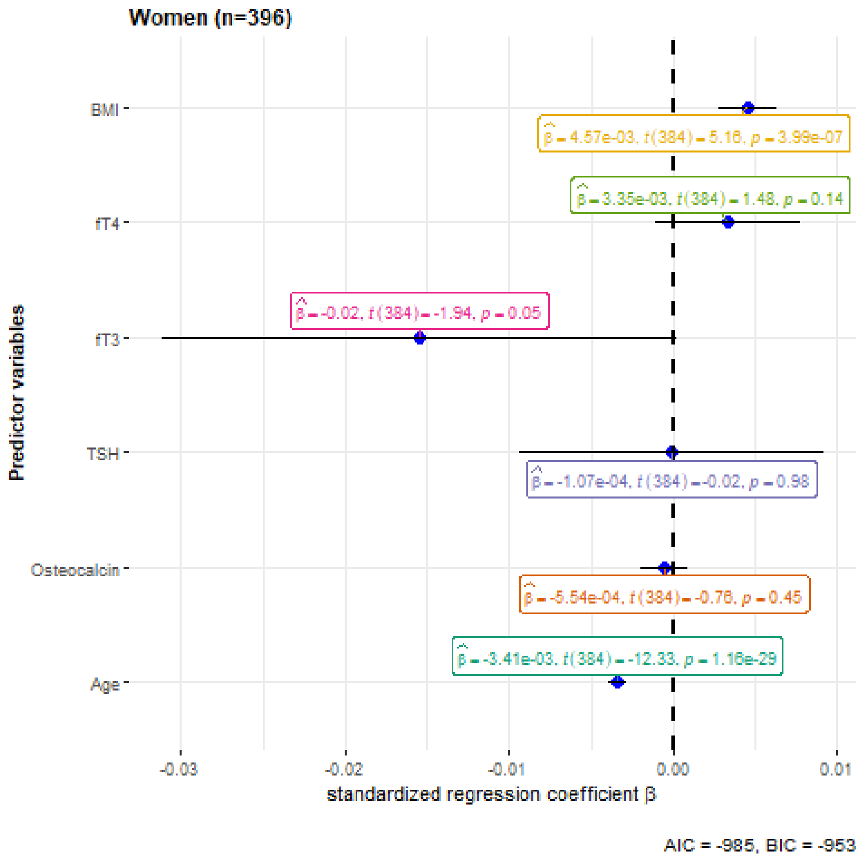

Figure S5. Effect sizes and 95% confidence intervals obtained from the linear regression analysis for the association of age, Osteocalcin, TSH, fT3, fT4 and BMI with BMD in the group of women (n=396). Statistically significant predictor variables are the lines that do not intersect with the vertical line at 0. If the lower limit of the confidence interval is above 0, the variable is positively associated with BMD, whereas if the upper limit of the confidence interval is below 0, the association is negative.

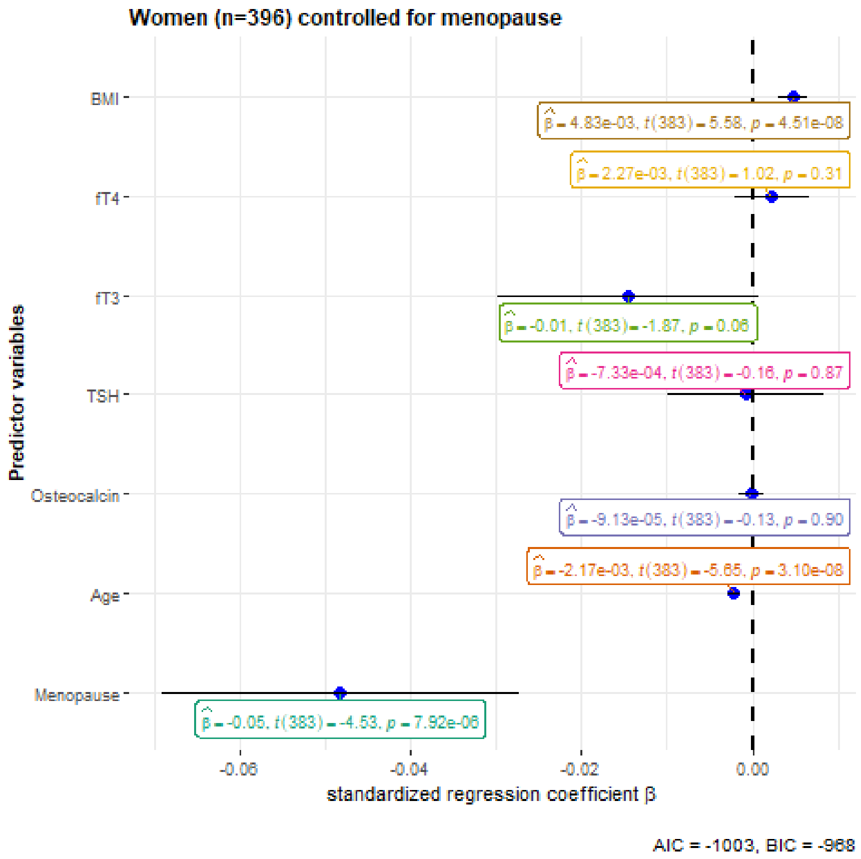

Figure S6. Effect sizes and 95% confidence intervals obtained from the linear regression analysis for the association of menopause status, age, Osteocalcin, TSH, fT3, fT4 and BMI with BMD in the group of women (n=396). Statistically significant predictor variables are the lines that do not intersect with the vertical line at 0. If the lower limit of the confidence interval is above 0, the variable is positively associated with BMD, whereas if the upper limit of the confidence interval is below 0, the association is negative.
